# Supplementary material for: Single-Cell RNA Sequencing of a Postmenopausal Normal Breast Tissue Identifies Multiple Cell Types That Contribute to Breast Cancer
Source: Cancers (Basel). 2020 Dec 4;12(12):3639. doi: 10.3390/cancers12123639 (PMC7761899; doi:10.3390/cancers12123639)
Supplement: Supplementary file 1 [file cancers-12-03639-s001.zip › cancers-989935-supplementary-xml/cancers-989935-supplementary.docx]

**Figure S1.** Monocle pseudotemporal trajectory analysis of mesenchymal cell types. (**A**) Clusters 5 (proposed mesenchymal progenitors) mapped to the branching point between endothelial (Cluster 2, 4), fibroblast (Cluster 3, 6), and smooth muscle (cluster 0) cells. The trajectory suggests potential cell differentiation relationships from Cluster 1 to 4 and from Cluster 6 to 3. Whether these contiguous relationships point to different cell states requires further analyses, (**B**) pseudotime shown in a gradient color from dark to light with Cluster 5 at the root.


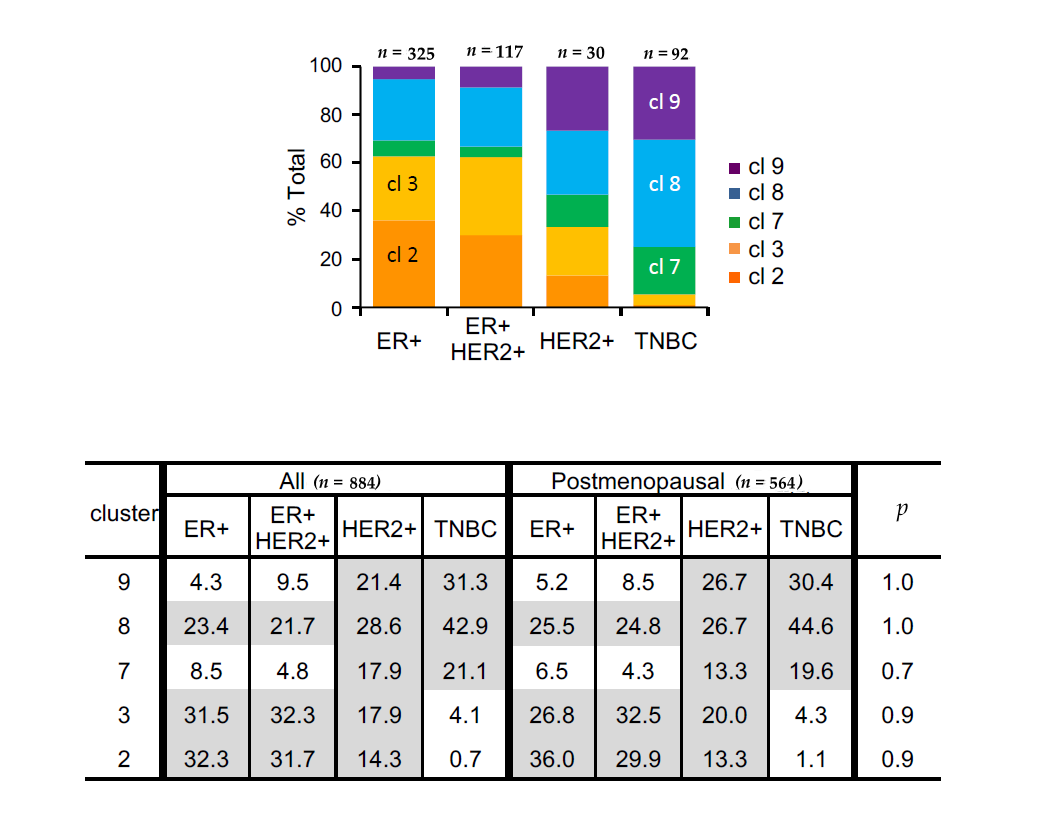


**Figure S2.** Postmenopausal patient tumors matching to single cell clusters in the TCGA data set. Cluster 2 and 3 signatures were prevalent in the ER+ tumors while Cluster 7 and 9 signatures were in the triple negative breast cancer (TNBC) subtype. Percentage of each tumor type is listed in the table for all tumors (left, *n* = 884) and postmenopausal patient tumors (right, *n* = 564). There were no differences in cell cluster type tumor distribution between all vs postmenopausal patient tumors (*p* values > 0.5). p values were calculated using student t-test.
